# Medicine at Home and Abroad

**Published:** 1953

**Authors:** John Apley

**Affiliations:** Consultant Paediatrician, United Bristol Hospitals and United Bath Hospitals


### MEDICINE AT HOME AND ABROAD

BY

JOHN APLEY, M.D., M.R.C.P.

*Consultant Paediatrician, United Bristol Hospitals and United Bath Hosp*

The Briton abroad who demands porridge, bacon, marmalade and te breakfast may never realize what he has missed; his loss cannot be expr merely in terms of croissants, black cherry jam and coffee. Meals are no joyed only at the dining-table; there are, indeed, banquets at which the dients are ideas, the courses are thoughts, and the waiters are words.

It is not only our own compatriots abroad but, equally, visitors to this cou who tend to measure everything by the hallowed yardstick of their na customs; and, of course, always with the naïve assumption that one's ow and one's own stick are inviolable. I remember being told, on returning fr visit to some children's hospitals in Barcelona, that we have nothing whate learn from Spain, apart from guitar playing and bull-fighting; my informa course, has yet to go there to confirm this opinion. It was amusing to set a this the remark of a worthy but equally untravelled Spanish physician wh the sort of occasion when thoughts are uninhibited and tongues loosened, re to my gambit on English medicine: " Well, Britain *is* an island; and isla *are* insular ".

Though I have often tried, I find it impossible to assess the impression visitors to this country take away from any centre of English medicine incidentally, is it not a little disturbing that so few do come here, except fro Commonwealth countries? But then, what would we like our visitors member, in Bristol, for example? Perhaps it is easier to be certain of the we would like them to forget, or never to have seen. We have, there is no at all, many things to be proud of: but what could visitors think of the garden at Dr. X's out-patient clinic; or of Mr. Y's surgical spelaeology theatre; or of Z's " research ", which we may know only too well is little than a form of occupational therapy for the medically sterile? We tend nat though perhaps subconsciously, to shepherd foreign lambs. When we abroad professionally we too are shepherded: we are led to graze in sel pastures, and we must be mindful not to bleat too dogmatically when we r to our own fold.

With this warning in mind it is, I think, instructive and certainly interest try to compare the standard of medicine, and for myself particularly of p trics, in our own and foreign countries. My own practice when I go abroa ask not only the natives but also other foreign visitors for their views: in th a wider perspective is obtained, though this " random sampling " is obv open to criticism, and what I choose to regard as a healthy English impar may be called by other names. I have tried this gambit in reverse at hom ive often been tempted to use a sphygmomanometer, or even a thermometer, 1 my respected colleagues when they accept it, as generally they do with pug-icious alacrity. In the remarks which follow most of the views expressed are ose of doctors discussing countries other than their own; though in some stances, of course with a sense of honest indignation, my own opinions insist oon bursting forth.

Thus, it still surprises me to hear so often that in clinical meeicine, an art on hich we modestly pride ourselves, other countries consider themselves our iperiors. In fact, there must be scarcely a country in the world whose doctors not believe sincerely that they themselves are the outstanding exponents of edside medicine. It is not in these islands alone that discussion is confined to ho is *second* best. From what I have gleaned, international opinion would vard the palm for clinical ability to the French. With the widespread admira-on for French diagnostic acumen there is, however, some deprecation of their ientific approach. Moreover, few doctors outside France approve of the poly-ierapeutic methods in vogue there, which seem inconsistent with the exquisite ecision of thought and expression associated with the French. " Just read the lvertisements in La Presse Medicale " I have heard said, justifiably, in scathing ristolian professorial tones; a remark followed by the characteristically honest lmission that those in the B.M.J. are almost as deplorable. Paris, which I cently visited with, if I may use the phrase, a " perambulation of paedia-icians ", illustrates the world-wide transition between the old and the new in edicine. In medical buildings, as in medical philosophy, there is much patching ancient structures. In a few cases there has been complete replacement, con-rming with the most modern standards; though whether these will survive as ng as their predecessors is doubtful. In France, as in other countries, I have een depressed rather than impressed, on seeing the latest productions, by a niformity which transcends international boundaries; this applies to new ospitals, units for premature infants, medical papers and even thoughts—just it does nowadays to tanks and tennis. The loss of national individuality leads ievitably to drabness, but perhaps it is good for us.

For a long time German medicine, with its strict but possibly excessively theo-etical discipline, was in the forefront of medical progress, but for many well nown reasons it has lost its proud position. Countries like Switzerland still laintain and add lustre to the German tradition, though the Swiss have been iticized because so many of their medical departments are subsidized by phar-iacological manufacturers. (Some Professorial Chairs in Switzerland, it has been id, are supported not by the traditional pillars of medicine, but by ampoules of ynthetic drugs).

It is widely accepted that the mantle of the Germans has now fallen on the mericans. In many illustrious centres in the U.S.A. the standards of medical ractice and research are, I agree wholeheartedly, unsurpassed anywhere in the orld, and great men and bold pioneers abound. But it must be admitted that here is often a startling gap between them and the more remote units in that ast and heterogeneous continent—a gap which is also evident in their equally ast and heterogeneous medical journals. Most European doctors who read ournals in the English language take North American ones in preference to ritish, though by no means accepting uncritically all that appears in them. The terms " mechanistic ", " technological " or " luxury " medicine are freq[illegible] applied; and a process of transmutation seems to be essential for countries [illegible] an older tradition (or a dollar-gap). It is not only in medicine that finance [illegible] part in moulding the underlying philosophy: wealthy countries like the [illegible] and Sweden are indulging increasingly in elaborate equipment, and it [illegible] some concern that this trend may lead to disuse atrophy of clinical craftsma[illegible] and judgment.

Sweden takes a justifiable pride in its abundance of first-class engineers [illegible] join their medical colleagues in perfecting ingenious and complicated app[illegible] Though this is undoubtedly helpful in promoting some forms of research [illegible] have the unfortunate effect of making the field workers and general practit[illegible] diffident and not self-reliant. Nevertheless, in Sweden I met outstandin[illegible] who have greatly advanced medicine, without the aid of machines and [illegible] appearing pedestrian. In Norway, doctors have had to keep pace without [illegible] thing but the simplest equipment; for Norway is as poor as Sweden is prosp[illegible] and medicine is perforce utilitarian, and attractively simple.

Spain similarly suffers the disadvantages and enjoys the independen[illegible] poverty. Despite the intense and very attractive individualism of the Sp[illegible] Spanish medicine still looks towards Germany, even in the neap tide of the [illegible] man tradition. There has always been a concentration on theoretical rather [illegible] practical instruction in Spanish medical schools, possibly due in part to the [illegible] Arabian influence in Spanish history; but the alert and agile-minded Sp[illegible] doctors very quickly remedy this defect in practice. In their acquaintance [illegible] world medicine those I met were impressively knowledgeable, though, be[illegible] of native difficulties, they have little opportunity for waging more than a g[illegible] type of warfare against disease. Large co-operative enterprises and public [illegible] work are comparatively deficient, and doctoring is hindered to some extent [illegible] paucity of nursing organizations which are independent of religious esta[illegible] ments.

In preventive medicine figures may speak more clearly than words, a[illegible] accompanying table shows the infant mortality rate and the total death rate [illegible] countries I have discussed.*

| | Infant Mortality per 1000 live births | Death Rate per 1000 inhab[illegible] |
|---|---|---|
| France | 55 | 13.5 |
| Switzerland | 34 | 10.7 |
| U.S.A. | 31 | 9.7 |
| Canada | 43 | 9.2 |
| Sweden | 23 | 9.9 |
| Norway | — | 8.9 |
| Spain | 68 | 11.3 |
| England and Wales | 32 | 11.7 |

* These figures apply to the year 1949, and are the latest available. They were [illegible] provided by Dr. S. W. Hinds, Lecturer in Child Health and Social Medicine, [illegible] University will be kept in mind that these figures reflect social and other factors as well the standard of medicine. It does, however, appear clear that England is one the safer places to be born in, though possibly it is not equally commendable r those who value longevity.

Of English medicine it is, I think, widely accepted abroad that one branch is e-eminent, to wit, anaesthesia—a point of view which will probably surprise me of us here. Tribute is paid to the English gift for clear clinical description, d admiration is expressed for the physiological background of English medical ork. I believe that the trend of English paediatrics from the hospital to the me is beginning to make a real impression on foreign observers. Perhaps it presses them all the more as an example of our adaptability, since the whole emendous field of social medicine is being cultivated here to some extent beuse of our lack of money, equipment and new buildings.

The subject of research is a difficult one to assess, partly because both quantity d quality have to be taken into account. However, in medical as in other forms investigation, I think it may be accepted that English fundamental research is tstanding, even though its practical application may sometimes lag behind.

rom the little I have learned about general practice abroad I have the impression at the average level here is at least as high as anywhere else, and probably is gher than in most countries.

Let me conclude by quoting two distinguished medical foreigners. The rst is the very eminent Dr. A. Blalock, who was in Bristol recently. He said he as glad to see signs that British medicine was, at last, losing its " inferiority mplex ". The other I quote anonymously (by request) but verbatim: " As for ritish medicine, somehow it has never attracted much attention. It is condered to be sound and conservative and commands respect but not worship.

ossibly the wider modern interest for medical literature in the English language ay subsidiarily (for the primary interest is in American papers) bring about a ider recognition of the excellent qualities of your seasoned yet modern concept medicine ". Even those who may not entirely hold with this statement will gree that a foreigner who writes English as fluently as this has a claim to our tention.

Should any foreign medical men happen, by some chance, to read this collecon of random thoughts they will doubtless accuse me of bias and insularity. I

ill affirm, nevertheless, that if I myself were a patient I should wish to be tended this country rather than any other. I trust my colleagues will make a note of is for future reference.

